# Urogenital *Chlamydia trachomatis* Infections among Ethnic Groups in Paramaribo, Suriname; Determinants and Ethnic Sexual Mixing Patterns

**DOI:** 10.1371/journal.pone.0068698

**Published:** 2013-07-17

**Authors:** Jannie J. van der Helm, Reinier J. M. Bom, Antoon W. Grünberg, Sylvia M. Bruisten, Maarten F. Schim van der Loeff, Leslie O. A. Sabajo, Henry J. C. de Vries

**Affiliations:** 1 STI Outpatient Clinic, Public Health Service Amsterdam, Amsterdam, The Netherlands; 2 Department of Research, Public Health Service Amsterdam, Amsterdam, The Netherlands; 3 Public Health Laboratory, Public Health Service Amsterdam, Amsterdam, The Netherlands; 4 Lobi Foundation, Paramaribo, Suriname; 5 Department of Experimental Virology, Academic Medical Center (AMC), University of Amsterdam, Amsterdam, The Netherlands; 6 Center for Infection and Immunity Amsterdam (CINIMA), Academic Medical Center, University of Amsterdam, Amsterdam, The Netherlands; 7 Dermatological Service, Ministry of Health, Paramaribo, Suriname; 8 Department of Dermatology, Academic Medical Center, University of Amsterdam, Amsterdam, The Netherlands; 9 Center for Infectious Disease Control, National Institute of Public Health and the Environment, Bilthoven, The Netherlands; University of California, San Francisco, University of California, Berkeley, and the Children’s Hospital Oakland Research Institute, United States of America

## Abstract

**Background:**

Little is known about the epidemiology of urogenital *Chlamydia trachomatis* infection (chlamydia) in Suriname. Suriname is a society composed of many ethnic groups, such as Creoles, Maroons, Hindustani, Javanese, Chinese, Caucasians, and indigenous Amerindians. We estimated determinants for chlamydia, including the role of ethnicity, and identified transmission patterns and ethnic sexual networks among clients of two clinics in Paramaribo, Suriname.

**Methods:**

Participants were recruited at two sites a sexually transmitted infections (STI) clinic and a family planning (FP) clinic in Paramaribo. Urine samples from men and nurse-collected vaginal swabs were obtained for nucleic acid amplification testing. Logistic regression analysis was used to identify determinants of chlamydia. Multilocus sequence typing (MLST) was performed to genotype *C. trachomatis*. To identify transmission patterns and sexual networks, a minimum spanning tree was created, using full MLST profiles. Clusters in the minimum spanning tree were compared for ethnic composition.

**Results:**

Between March 2008 and July 2010, 415 men and 274 women were included at the STI clinic and 819 women at the FP clinic. Overall chlamydia prevalence was 15% (224/1508). Age, ethnicity, and recruitment site were significantly associated with chlamydia in multivariable analysis. Participants of Creole and Javanese ethnicity were more frequently infected with urogenital chlamydia. Although sexual mixing with other ethnic groups did differ significantly per ethnicity, this mixing was not independently significantly associated with chlamydia. We typed 170 *C. trachomatis*-positive samples (76%) and identified three large *C. trachomatis* clusters. Although the proportion from various ethnic groups differed significantly between the clusters (*P* = 0.003), all five major ethnic groups were represented in all three clusters.

**Conclusion:**

Chlamydia prevalence in Suriname is high and targeted prevention measures are required. Although ethnic sexual mixing differed between ethnic groups, differences in prevalence between ethnic groups could not be explained by sexual mixing.

## Introduction

Urogenital *Chlamydia trachomatis* infection, or chlamydia, is the most prevalent bacterial sexually transmitted infection (STI) worldwide [1]. Left untreated, chlamydia can lead to complications like pelvic inflammatory disease, ectopic pregnancy, and infertility. To reduce complications and transmission of chlamydia, active case finding and early treatment are critical strategies [2], [3]. Suriname is on the South American continent, but as a consequence of a shared colonial past it is more socio-culturally connected to the Caribbean region. The prevalence of chlamydia in the general population in many countries of the Caribbean is unknown because testing facilities are lacking and routine screening is not available. A study in Guadeloupe among patients who were referred for a genital infection, showed a prevalence of 17% among men and 10% among women [4]. A study in Barbados among the general population showed a prevalence of 11% [5] and a study in Trinidad and Tobago among pregnant women showed a prevalence of 21% [6]. We previously found a prevalence of 21% among high-risk women and 9% among low-risk women in Suriname [7].

The variety of ethnicities is distinctive for Surinamese society. The Surinamese population consists of Creoles and Maroons (both descendants of African diaspora due to the slave trade), Hindustani, Javanese, and Chinese (all descendants of labor immigrants from the former British Indies, Dutch Indies, and China, respectively), Caucasians (descendants of European colonialists), indigenous Amerindians, and people of mixed race. The five major groups are Hindustani (27.4%), Creole (17.7%), Maroon (14.7%), Javanese (14.6%), and mixed race (12.5%). These groups cannot be considered a ‘minority’ since they are comparable in size and integrated parts of the total population [8]. Previous Surinamese studies on sexuality, however, have mainly focused on the Creoles, and rarely on other ethnicities [9], [10].

The structure of sexual networks is important for STI transmission, but elucidating these transmission networks based on epidemiological and behavioral data alone is challenging. Combining epidemiological and behavioral data with molecular microbial genotyping techniques can provide more insight into the transmission patterns of *C. trachomatis*. Molecular typing can reveal the relatedness of bacterial strains that circulate among the population and may identify transmission networks at the pathogen level. Because of the low genetic variability of *C. trachomatis*, a typing tool with a high discriminatory resolution between strains is necessary to reveal network associations of *C. trachomatis*. Whereas suitable molecular techniques for *Neisseria gonorrhoeae* have been available for some time [11], high-resolution typing methods for *C. trachomatis*, such as multilocus sequence typing (MLST), have only been developed recently [12], [13]. Studies using high-resolution typing of *C. trachomatis* strains have examined the relation between clinical symptoms [14], geographic location [15], and sexual risk group [16], [17]. The relation between ethno-demographic characteristics and *C. trachomatis* strains in sexual transmission networks of heterosexual populations has not yet been analyzed using high-resolution molecular pathogen typing.

Earlier we reported a high chlamydia prevalence among both low- and high-risk women [7]. Here we report on the chlamydia prevalence among women as well as men. The aim of our study among men and women at two clinics in Paramaribo, Suriname was to elucidate determinants for chlamydia, notably the role of ethnicity and ethnic sexual mixing, and to identify transmission patterns and sexual networks using molecular epidemiological network analyses.

## Methods

### Ethics Statement

The study was approved by the ethics committee of the Ministry of Health of the Republic of Suriname (VG010-2007) and the ethics committee of the Academic Medical Center, University of Amsterdam, the Netherlands (MEC07/127). Patients participated anonymously and gave written informed consent.

### Recruitment Sites and Population

Participants were recruited at two sites in Paramaribo, Suriname:

1. The Dermatological Service, an integrated outpatient STI clinic, frequented by men and women, that offers free-of-charge examination and treatment of STIs and infectious skin diseases such as leprosy and leishmaniasis,. All individuals who visited for an STI check-up were invited to participate in the study. These participants were considered to be a ‘high-risk’ population for chlamydia.
2. The Lobi Foundation, a family planning (FP) clinic frequented by women only. All consecutive women visiting the clinic were invited to participate in the study. As women do not primarily visit this clinic to be checked for STIs, these participants were considered to be a ‘low-risk’ population for chlamydia.

Recruitment took place between March 2008 and July 2010. Exclusion criteria were: age younger than 18 years and previous participation in the study. A nurse interviewed participants about demographic characteristics (including self-reported ethnicity) and sexual behavior.

### Specimen Collection and Testing Procedures

Urine samples from males and nurse-collected vaginal swabs from females were obtained for nucleic acid amplification test (NAAT) testing with the monospecific Aptima Chlamydia assay for the detection of *C. trachomatis* rRNA (Hologic Gen-Probe Inc., San Diego, USA). Nurses were trained to collect the swabs before routine speculum examination was performed, as described before [7]. The samples were collected according to the manufacturer’s instructions, stored in a fridge (at temperature between 2° and 7°C) and packed according to IATA rules for transport by plane to the Public Health Laboratory in Amsterdam for NAAT testing. Technicians performing NAAT did not receive any information about the participant. NAAT test results were forwarded to the two clinics in Suriname, where the chlamydia positive participants were treated within 1 to 8 weeks after the clinic visit with doxycycline (100 mg bid for 7 days at the FP clinic and 100 mg bid for 10 days at the STI clinic) or, in case of (probable) pregnancy, with a single 1000 mg oral dose of azithromycin. Participants who tested positive for urogenital chlamydia also received treatment to be used by their partner(s).

### MLST

Multilocus sequence typing (MLST) was used to genotype *C. trachomatis*. Details of this method were described previously [13]. In brief, DNA was extracted at the Public Health Laboratory Amsterdam from transport medium in which the swab or urine had been put, using isopropanol precipitation. All DNA isolates were tested for the presence of chlamydial DNA with the in-house *pmpH* qPCR as described previously [18], [19]. The DNA isolates were amplified by a nested PCR and sequenced for the regions *ompA*, CT046 (*hctB*), CT058, CT144, CT172 and CT682 (*pbpB*). The sequences were checked against an in-house library and against the *Chlamydia trachomatis* MLST database (mlst.bmc.uu.se), and were given an allele number for each region. Only samples of which all alleles were successfully amplified, sequenced and identified, and therefore had obtained a full MLST profile (sequence type, ST), were included in the analyses. As *ompA* is part of the MLST scheme, genovars could be assigned for all included samples. A minimum spanning tree was generated using MLST profiles. Cluster analysis was performed allowing single locus variance using BioNumerics 7 (Applied Maths, Sint-Martens-Latem, Belgium). A cluster was defined as a group of STs differing by not more than one locus from another ST within that group, and had to include at least 10% of the total number of samples (i.e. at least 17 samples). Typing data of the study population are also reported in a paper comparing the distributions of *C. trachomatis* strains among residents of Paramaribo and residents of Amsterdam (Bom *et al*., submitted).

### Statistical Analysis

The study population consisted of high-risk men and women recruited at the STI clinic and low-risk women recruited at the FP clinic. To examine whether epidemiological characteristics differed between these three study groups the χ^2^-test for independence was used. Prevalence was calculated as the number of positive tests in the study period divided by the total number of individuals tested in the study period [20]. To assess determinants of chlamydia, we performed univariable logistic regression analysis and examined the effect of the following variables: age, education, ethnicity, different ethnic group of sexual partners (i.e. ethnic sexual mixing), study group (including sex and recruitment site), condom use, number of partners in the preceding month, number of partners in the preceding 12 months, having had sex in exchange for money or goods, and (for men) having had sex with men (MSM). Age was divided into four categories. Because of small numbers we grouped Caucasian, Chinese, and Indigenous Amerindian ethnicities together in univariable and multivariable analyses. Ethnic sexual mixing was defined as having had sex with at least one partner of another ethnicity in the preceding 12 months. Variables that were associated with chlamydia at *P*≤0.1 in the univariable analysis were entered into a multivariable model. Higher chlamydia prevalence at younger age and, with a higher number of sexual partners has been established by various other studies [21] and therefore these determinants were forced into the multivariable model. Ethnicity and ethnic group of the partner(s) were variables of specific interest and were also forced into the model. To avoid multicollinearity, we only included the variable ‘number of partners in the preceding 12 months’ in the model and not the variable ‘number of partners in the preceding month’. We considered *P*<0.05 as statistically significant. We checked for interactions between study group and all other variables in the final model and also checked for interactions between the number of partners in the preceding 12 months and ethnic sexual mixing.

To examine whether ethnic group was a determinant for ethnic sexual mixing, we performed a multivariable logistic regression analysis. Variables that were associated with ethnic sexual mixing at *P*≤0.1 in univariable analysis were entered into a multivariable model. The final model included number of partners in the preceding 12 months, sex in exchange for money or goods, and study group.

We compared the observed frequency of people who had sexual partners from their own ethnicity with the expected frequency (if partner selection from the population would have occurred at random with respect to ethnic background) by using the χ^2^ goodness-of-fit test [22]. The expected number of people with sexual partners from their own ethnicity was calculated by multiplying the total number of reported partners of an ethnicity by the proportion of individuals from each ethnicity in the study. In order to identify transmission patterns and sexual networks, a minimum spanning tree was made with different colors for different ethnicities and *C. trachomatis* clusters were compared in terms of ethnic composition using χ^2^-tests for independence. Analyses were performed with SPSS package version 19.0 (SPSS Inc., Chicago, IL).

## Results

### Study Population

A total of, 415 men and 1093 women were included in the study. The response rate among men was 78.3%, among women visiting the STI clinic 83.0% and among women visiting the FP clinic 99.8%. The included and excluded men did not differ by age (*P* = 0.303) and ethnicity (*P* = 0.329). The included and excluded women visiting the STI clinic had a comparable age (*P* = 0.238) but ethnicity did differ (*P* = 0.020). Demographics and sexual behavior of study participants are shown in Table 1. All epidemiological characteristics differed significantly between the three study groups. The overall median age was 29 years (IQR 25–37) and the majority had a low (40.5%) or medium (42.9%) level of education. In total, 444 (29.4%) were of Creole ethnicity, 289 (19.2%) of Hindustani ethnicity, 177 (11.7%) of Javanese ethnicity, 277 (18.4%) were mixed race, and 258 (17.1%) were of Maroon ethnicity. Women visiting the STI clinic were younger compared with men from the same site and women visiting the FP clinic (*P*<0.001). Women visiting the STI clinic reported higher risk behavior, such as >2 partners in the previous year (23.0%), and more frequently reported sex in exchange for money or goods (16.7%), compared with women visiting the FP clinic (6.1% and 0.7%, respectively).

**Table 1:** Epidemiological characteristics of the study population by study group in Paramaribo, Suriname, 2008–2010.

		Men recruitedat STI clinic	Women recruitedat STI clinic	Womenrecruitedat familyplanningclinic	*P* value	Total studypopulation
		(N = 415)	(N = 274)	(N = 819)		(N = 1508)
		n (%)	n (%)	n (%)		n (%)
**Demographic characteristics**						
Median age in years (IQR)		29 (25–38)	28 (23–33)	31 (25–37)	<0.001	29 (25–37)
Age in years	<25	109 (26.3)	105 (38.3)	184 (22.5)	<0.001	398 (26.4)
	25–29	116 (28.0)	67 (24.5)	198 (24.2)		381 (25.3)
	30–34	59 (14.2)	47 (17.2)	173 (21.2)		279 (18.5)
	> = 35	131 (31.6)	55 (20.1)	264 (32.2)		450 (29.8)
Education	Low	235 (56.6)	99 (36.1)	277 (33.8)	<0.001	611 (40.5)
	Medium	111 (26.7)	109 (39.8)	427 (52.1)		647 (42.9)
	High	40 (9.6)	46 (16.8)	111 (13.6)		197 (13.1)
	Unknown	29 (7.0)	20 (7.3)	4 (0.5)		53 (3.5)
Ethnic group^a^	Caucasian	2 (0.5)	11 (4.0)	6 (0.7)	<0.001	19 (1.3)
	Chinese	1 (0.2)	6 (2.2)	6 (0.7)		13 (0.9)
	Creole	166 (40.0)	79 (28.8)	199 (24.3)		444 (29.4)
	Hindustani	33 (8.0)	30 (10.9)	226 (27.6)		289 (19.2)
	Indigenous	6 (1.4)	9 (3.3)	10 (1.2)		25 (1.7)
	Javanese	14 (3.4)	17 (6.2)	148 (17.9)		177 (11.7)
	Maroon	120 (28.9)	53 (19.3)	85 (10.4)		258 (17.1)
	Mixed	72 (17.3)	67 (24.5)	138 (16.8)		277 (18.4)
**Sexual behavior**						
Ethnic sexual mixing^a^	Had only sexual partnersfrom same ethnic group	162 (40.5)	120 (48.0)	508 (64.9)	<0.001	790 (55.1)
	Had at least one sexual partnerfrom another ethnic group	238 (59.5)	130 (52.0)	275 (35.1)		643 (44.9)
Condom use^a^	Always	126 (30.7)	76 (28.0)	79 (9.8)	<0.001	281 (18.8)
	Never or inconsistent	285 (69.3)	195 (72.0)	731 (90.2)		1211 (81.2)
Number of partners preceding month^a^	0	22 (5.3)	16 (6.3)	34 (4.2)	<0.001	72 (4.9)
	1	228 (55.1)	185 (73.4)	741 (91.7)		1154 (78.3)
	>1	164 (39.6)	51 (20.2)	33 (4.1)		248 (16.8)
Median number of partners in thepreceding 12 months (IQR)		2 (1–4)	1 (1–2)	1 (1–1)	<0.001	1 (1–2)
Mean number of partners in thepreceding 12 months		7	16	1		
Number of partners in thepreceding 12 months	0	2 (0.5)	11 (4.0)	11 (1.3)	<0.001	24 (1.6)
	1	109 (26.3)	136 (49.6)	649 (79.2)		894 (59.3)
	2	114 (27.5)	64 (23.4)	108 (13.2)		286 (19.0)
	>2	190 (45.8)	63 (23.0)	51 (6.1)		304 (20.2)
Sex in exchange for money or goods^a^		11 (2.7)	45 (16.7)	6 (0.7)	<0.001	62 (4.2)
Men having sex with men^a^		7 (1.7)	NA	NA		7 (1.7)
**Chlamydia prevalence**						
*Chlamydia trachomatis* infectiondiagnosis by NAAT		95 (22.9)	51 (18.6)	78 (9.5)	<0.001	224 (14.9)

a Numbers do not add up to the column total due to missing data, percentages do add up to 100%.

Missing data: ethnic group n = 6, ethnic sexual mixing n = 75, condom use n = 16, number of partners in the preceding month n = 34, sex in exchange for money or goods n = 22, men having sex with men n = 3.

IQR, interquartile range; NA, not available; NAAT, nucleic acid amplification test; p-values based on men attending the STI clinic, women attending the STI clinic and women attending the family planning clinic.

### Prevalence and Determinants of Chlamydia

The prevalence of chlamydia was 18.6% (95% CI, 14.3–23.6%) among women visiting the STI clinic, 9.5% (95% CI, 7.7–11.7%) among women visiting the FP clinic [7] and 22.9% (95% CI, 19.0–27.1%) among male STI clinic visitors. The highest prevalence of chlamydia was found among Creole men visiting the STI clinic (30.1%) but this was not significantly higher than the prevalence among men from other ethnic groups which ranged between 15.2% and 21.4% (*P* = 0.123). Hindustani women had a slightly lower prevalence (6.3%) compared with women from other ethnic groups, which ranged between 10.9% and 15.3% (*P* = 0.054). Univariable associations between epidemiological characteristics and chlamydia are shown in Table 2. Age, ethnic group, ethnic sexual mixing, study group and number of partners in the preceding 12 months were significantly associated with chlamydia in univariable analysis. In multivariable analysis, chlamydia was significantly associated with ethnic group (OR, 1.76; 95% CI, 1.03–3.00 for Creoles, OR, 2.05; 95% CI, 1.09–3.84 for Javanese, both compared with Hindustani); age (OR, 3.01; 95% CI, 1.93–4.71 for those aged <25 years, compared with those aged ≥35); and study group (OR, 2.30; 95% CI, 1.52–3.49 for men visiting the STI clinic and OR, 1.91; 95% CI 1.24–2.94 for women visiting the STI clinic, both compared with women visiting the FP clinic), but not with ethnic sexual mixing (OR, 1.33; 95% CI, 0.96–1.85) and number of partners in the preceding 12 months (OR, 1.39; 95% CI, 0.92–2.11 for having >2 partners compared with having ≤1 partner) (Table 2).

**Table 2:** Univariable and multivariable logistic regression analyses of determinants associated with chlamydia among the study population included at two sites in Paramaribo, Suriname, 2008–2010.

		NAATpositive	UnivariableOR (95%CI)	*P* value	MultivariableAdjusted OR(95%CI)*	*P* value
		224/1508 (14.9)				
		n/N (%)				
Study group	Family planning clinic – women	78/819 (9.5)	1	<0.001	1	<0.001
	STI clinic – women	51/274 (18.6)	2.17 (1.48–3.19)		1.91 (1.24–2.94)	
	STI clinic – men	95/415 (22.9)	2.82 (2.03–3.91)		2.30 (1.52–3.49)	
Age in years	<25	90/398 (22.6)	3.36 (2.22–5.08)	<0.001	3.01 (1.93–4.71)	<0.001
	25–29	71/381 (18.6)	2.63 (1.72–4.04)		2.60 (1.65–4.09)	
	30–34	27/279 (9.7)	1.23 (0.73–2.08)		1.26 (0.73–2.18)	
	> = 35	36/450 (8.0)	1		1	
Education	Low	96/611 (15.7)	1	0.878		
	Medium	93/647 (14.4)	0.90 (0.66–1.23)			
	High	27/197 (13.7)	0.85 (0.54–1.35)			
	Unknown	8/53 (15.1)	0.95 (0.44–2.09)			
Ethnic group	Creole	87/444 (19.6)	3.11 (1.88–5.14)	0.001	1.76 (1.03–3.00)	0.027
	Hindustani	21/289 (7.3)	1		1	
	Javanese	28/177 (15.8)	2.40 (1.32–4.37)		2.05 (1.09–3.84)	
	Maroon	36/258 (14.0)	2.07 (1.17–3.65)		0.96 (0.52–1.78)	
	Mixed	44/277 (15.9)	2.41 (1.39–4.17)		1.33 (0.72–2.35)	
	Other^a^	7/57 (12.3)	1.79 (0.72–4.43)		1.01 (0.38–2.67)	
Ethnic sexual mixing	Had only sexual partnersfrom same ethnic group	88/790 (11.1)	1	<0.001	1	0.090
	Had at least one sexual partnerfrom another ethnic group	125/643 (19.4)	1.93 (1.43–2.59)		1.33 (0.96–1.85)	
Condom use	Always	45/281 (16.0)	1	0.529		
	Never or inconsistent	176/1211 (14.5)	0.89 (0.62–1.27)			
Number of partners in thepreceding month	0	8/72 (11.1)	1	<0.001		
	1	152/1154 (13.2)	1.21 (0.57–2.58)			
	>1	58/248 (23.4)	2.44 (1.11–5.39)			
Number of partners in thepreceding 12 months	≤1	104/918 (11.3)	1	<0.001	1	0.225
	2	49/286 (17.1)	1.62 (1.12–2.34)		1.33 (0.88–2.01)	
	>2	71/304 (23.4)	2.39 (1.71–3.33)		1.39 (0.92–2.11)	
Sex in exchange formoney or goods	No	207/1424 (14.5)	1	0.166		
	Yes	13/62 (21.0)	1.56 (0.83–2.93)			

* ORs in the multivariable model are adjusted for all factors for which adjusted ORs are shown.

a Other: Caucasian, Chinese, Indigenous.

NAAT, nucleic acid amplification test; OR, odds ratio; 95%CI, 95% confidence interval.

The interactions between study group and all other variables in the final model and the interaction between the number of partners in the preceding 12 months and ethnic sexual mixing were not significant.

### Sexual Mixing among Ethnic Groups

A total of 643 participants (43.6%) reported sexual mixing with other ethnic groups, and 790 (52.4%) did not report any sexual mixing. Ethnic sexual mixing differed between ethnic groups. Of the Hindustani 65 (23.5%) reported sexual mixing. This was higher for individuals with Creole (n = 191; 44.3%), Javanese (n = 85; 49.4%), Maroon (n = 95; 38.6%), or mixed race (n = 170; 65.9%) ethnicity (*P*<0.001). In multivariable analysis, adjusting for number of partners in the preceding 12 months, sex in exchange for money or goods, and study group, ethnic sexual mixing was significantly associated with ethnic group (OR, 1.87; 95% CI, 1.30–2.70 for Creoles; OR, 3.34; 95% CI, 2.18–5.11 for Javanese; OR, 1.15; 95% CI, 0.75–1.76 for Maroon; OR, 4.88; 95% CI, 3.26–7.30 for mixed race; all compared with Hindustani).

Table 3 shows the ethnic groups of the participants included in the study and the observed and expected ethnic background of their partners. Of the Creole, Hindustani, Javanese and Maroon participants between 60.5% and 77.9% reported to have had sex with a partner of their own ethnicity; for mixed race individuals this was 44.4%. Maroon individuals were more likely to have a partner with a Creole ethnicity (29.8%) compared with a partner with a Hindustani (3.1%) or Javanese ethnicity (3.9%). Likewise, only 1% of the Hindustani and Javanese individuals reported sex with a Maroon partner. The observed frequencies of only having sexual partners from participants’ own ethnicity were significantly higher than expected frequencies if partners had been selected from the population at random with respect to ethnicity (*P*<0.001 for all 5 major ethnic groups).

**Table 3:** Ethnic sexual mixing patterns of men and women, Paramaribo, Suriname, 2008 to 2010.

	Ethnic group of sexual partner									
	Creole partner		Hindustani partner		Javanese partner		Maroon partner		Mixed race partner	
	O	E	O	E	O	E	O	E	O	E
	n (%)		n (%)		n (%)		n (%)		n (%)	
**Ethnic group of study participants**										
Creole (n = 444)	**314 (70.7)**	**157**	26 (5.9)	104	29 (6.5)	60	51 (11.5)	80	99 (22.3)	99
Hindustani (n = 289)	20 (6.9)	103	**225 (77.9)**	**68**	14 (4.8)	39	3 (1.0)	52	32 (11.1)	65
Javanese (n = 177)	12 (6.8)	63	28 (15.9)	41	**107 (60.5)**	**24**	1 (0.6)	32	43 (24.3)	40
Maroon (n = 258)	77 (29.8)	91	8 (3.1)	60	10 (3.9)	35	**196 (76.0)**	**47**	25 (9.7)	58
Mixed (n = 277)	93 (33.6)	98	53 (19.1)	65	39 (14.1)	37	17 (6.1)	50	**123 (44.4)**	**62**
Other (n = 63)^a^	19 (30.2)	22	13 (20.6)	15	4 (6.3)	9	4 (6.3)	11	16 (25.3)	14
**Tota**l	535	534	353	353	203	204	272	272	338	338
**O/E**	2.0		3.3		4.5		4.2		2.0	
	?^2^ = 268, p<0.001		?^2^ = 472, p<0.001		?^2^ = 339, p<0.001		?^2^ = 585, p<0.001		?^2^ = 96, p<0.001	

Percentages in row totals can exceed 100% as participants may have partners from various ethnicities.

O = observed; E = expected. χ^2^ based on goodness of fit.

The expected number of people with sexual partners from their own ethnicity was calculated by multiplying the total number of reported partners of an ethnicity (e.g. n = 353 for Hindustani) by the proportion of individuals from each ethnicity in the study (e.g. 19% for Hindustani). O/E is the ratio of the observed number of partners (e.g. n = 225 for Hindustani) divided by the expected number of partners from that ethnic group (e.g. n = 68 for Hindustani).

a Other; Caucasian, Chinese, Indigenous (n = 57), unknown (n = 6).

### Genovar Typing and MLST

We were able to type 170 samples of the 224 *C. trachomatis* positive samples (75.9%). The strains belonged to nine *ompA* genovars, predominantly E (32.4%), F (19.4%), D (18.2%) and I (12.9%). Furthermore, J (5.9%), G (5.3%), K (2.9%), B (1.8%), and H (1.2%) were found.

**Figure 1:**
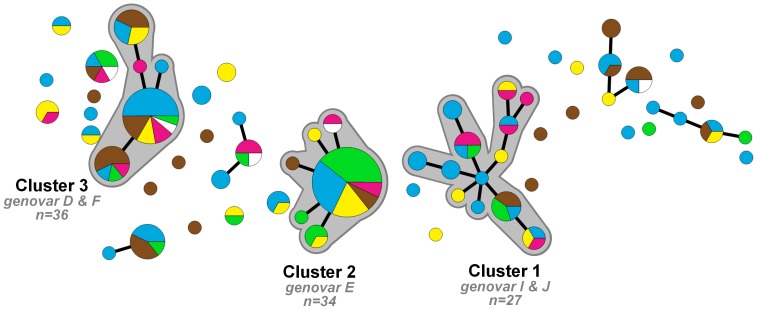
Minimum spanning tree of 170 *Chlamydia trachomatis* positive samples in Paramaribo, Suriname 2008–2010. Each circle represents one MLST type. Size of the circles is proportional to the number of identical MLST profiles. Bold lines connect types that differ by one single locus. Halos indicate the three large distinct clusters (≥27 samples). Colors indicate ethnicity; blue – Creole, brown – mixed race, green – Javanese, yellow – Maroon, pink – Hindustani, white – Indigenous Amerindian, Caucasian and unknown.

Among the 170 fully typed samples, we identified 65 different MLST profiles of which 32 (49%) were novel when checked against the MLST database on January 8, 2013. These novel MLST profiles were found in 52 (31%) of 170 samples. A minimum spanning tree was generated using MLST profiles (Figure 1) in which three large distinct clusters of *C. trachomatis* strains could be identified. Cluster 1 consisted of 27 samples (genovars I (81.5%) and J (18.5%)), cluster 2 consisted of 34 samples (all genovar E) and cluster 3 consisted of 36 samples (genovars D (23.5%) and F (76.5%)). There were 13 smaller clusters (containing 2–10 samples) and 20 singletons.

**Figure 2:**
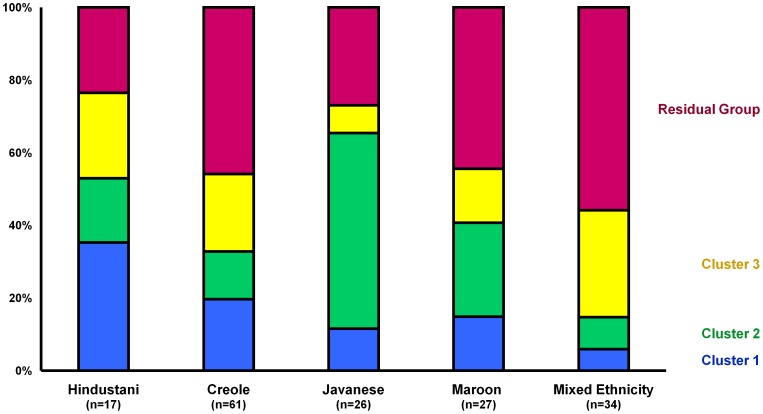
Distribution of individuals in *Chlamydia trachomatis* clusters within ethnic groups in Paramaribo, Suriname, 2008–2010. Colors indicate cluster: Blue – cluster 1; Green – cluster 2; Yellow – cluster 3; Pink – residual group.

Although all five major ethnic groups were represented in all three clusters, the proportion from various ethnic groups differed significantly between the three clusters (*P* = 0.003). Figure 2 shows the distribution of individuals in each cluster for each ethnic group. Individuals with Javanese ethnicity were mainly found in cluster 2 (53.8%). Of the Hindustani, 35.1% belonged to cluster 1. Of the Creole and mixed race individuals 45.9% and 55.9%, respectively, were found outside the three main clusters of *C. trachomatis* strains.

## Discussion

### Prevalence of Chlamydia in Suriname

This is the first report on epidemiology of chlamydia in Suriname in both a high-risk and a low-risk population. Previously, we described the high prevalence of 9% and 21% respectively among low-risk and high-risk Surinamese women [7]; here we report a very high prevalence of 23% among high-risk Surinamese men. The high prevalence found in Suriname in our study can partly be attributed to the current lack of screening facilities in Suriname. The prevalence among women visiting the FP clinic was comparable to that reported by a recent study in the Caribbean region among women in the general population [5]. Another study in the region among women who were referred for a genital infection, presumably a high-risk group for chlamydia, reported a prevalence of 11% [4], and among pregnant women a prevalence of 21% was found [6]. Studies among STI clinic visitors in the Caribbean region are scarce, but a study in Jamaica from 1999, in which chlamydia was tested by direct fluorescence assay and culture (which are now considered obsolete diagnostics), reported a prevalence of 55% [23]. The high prevalence found in low- or middle-income countries indicates the urgent need for reliable and affordable diagnostics, preferably a point-of-care test. The prevalence found among the STI clinic visitors is likely to be higher than the prevalence in the Surinamese general population. Furthermore, despite the high response rate, the included and excluded women visiting the STI clinic differed by ethnicity and the reported prevalence should be interpreted as the prevalence among those who were tested and might not reflect the ‘true’ prevalence. The response rate among the population attending the FP clinic was very high and the prevalence may be a reasonable reflection of the sexually active Surinamese population. The higher prevalence indicates that preventive measures focused on the sexually active population in general are urgently necessary. Our study showed that the younger age group was disproportionately affected by chlamydia, so targeting prevention at this group seems most cost-effective, especially since safe sex messages probably will be more effective at sexual debut [9].

### Prevalence of Chlamydia among Ethnic Groups

The Creole and Javanese groups seemed more affected by chlamydia compared with the Hindustani. A study from Trinidad and Tobago performed in 2004 compared three ethnic groups (African, East Indian and mixed race) using univariable analysis and found that individuals of East Indian descent were less likely to be infected with chlamydia compared with those of African descent [6]. Compared with Trinidad and Tobago, a society characterized by two dominant ethnic groups, Surinamese society is much more ethnically diverse. Since the prevalence in all but one ethnic group was above 12%, testing and treatment of all groups is required. The distribution of ethnic groups included in our study was approximating a correct representation of the actual Surinamese population according to the 2004 population census [8], although our study included more Creole and mixed race individuals and less people with Hindustani ethnicity.

### Chlamydia and Sexual Mixing among Ethnic Groups

Previously it was found that sexual mixing patterns could be important for dynamics of the spread of STI [24]. Here we show that the frequency of having only sexual partners from participants’ own ethnicity was higher than expected if partners would have been selected regardless of ethnicity (i.e. assortative mixing). On the other hand, almost half of the study population reported ethnic sexual mixing, which showed that bridges between the ethnic groups do exist. Ethnic sexual mixing differed per ethnic group and was highest for mixed race individuals followed by Javanese and Creoles. Nevertheless, this sexual mixing was not associated with chlamydia, which is in line with a study among immigrants in the Netherlands where ethnic sexual mixing was not associated with self-reported STI [25]. One study on ethnic mixing of Surinamese immigrants in the Netherlands showed that Hindu-Surinamese individuals were more likely to mix than Afro-Surinamese individuals [26], which is in contrast to our data derived from Suriname. In the Dutch study among Surinamese migrants, eight times more Afro-Surinamese individuals were included than Hindu-Surinamese individuals [26]. Population size and the size of the ethnic group are of importance for the likelihood of ethnic sexual mixing and might explain the difference between the studies. Hindu-Surinamese migrants in the Netherlands might be more likely to meet individuals from different ethnicities, and therefore more likely engage in ethnic sexual mixing.

Besides the epidemiological characterization of ethnic sexual mixing, we used MLST typing to identify pathogen-associated networks among participants with chlamydia. Previously *ompA* genovar typing has been used [27] but MLST was found to be more discriminative and provides more solid proof of independent circulation [17]. Furthermore MLST is less affected by intragenic recombination of *C. trachomatis*, than typing based on only the *ompA* gene is [28]. MLST revealed that all major ethnic groups were represented in all three clusters of *C. trachomatis* strains, which suggests that these strains circulate endemically among all ethnic groups. Although the distribution of clusters within each group varied, clear separate networks for *C. trachomatis* transmission by ethnicity could not be identified which can be explained by ethnic sexual mixing between the groups. The typing results were in agreement with the epidemiological data. In contrast, studies comparing non-mixing populations (heterosexuals and MSM) showed hardly any overlap in chlamydial strains [16], [17]. In our study we could not distinguish separate networks between MSM and heterosexuals as only 7 MSM were included of whom only one had chlamydia and the provided sample could not be typed by MLST.

By checking the MLST profiles in the international *C. trachomatis* database, 32 (49%) novel strains were found. This database has existed since 2007 and comprises 459 profiles to date. It is most likely that many novel strains will be found. The database will expand and include an increasingly wide variety of *C. trachomatis* strains from different parts of the world. Combining the molecular data with epidemiological characteristics will provide more insight into global *C. trachomatis* transmission networks.

Several potential limitations should be mentioned. In our study differences in prevalence of chlamydia between ethnic groups were found, but we were not able to elucidate why these differences exist between ethnic groups. Therefore, additional risk behavior data are necessary. For example, concurrency is an important factor in the spread of HIV/STI [29] and is not uncommon among Surinamese individuals [26], [30], [31]. Also, we did not have information on other partner characteristics besides ethnic group such as age, type of partner (regular or casual), or condom use with different partners. Mathematical transmission models could be of added value to better identify sexual networks. Participants were interviewed face to face by a research nurse. Therefore, they may have given socially desirable answers and response bias may have occurred. Such bias might have occurred for variables such as condom use and number of sexual partners and would probably have resulted in an underestimation of risk behavior. Furthermore, ethnicity of the partner was self-reported and misclassification cannot be excluded.

In conclusion, the prevalence of chlamydia in Suriname is very high, with 10% in a low-risk population and up to 23% in a high-risk population. This prevalence is high overall in all ethnic groups (>7%), but higher in the Creole and Javanese groups compared with the Hindustani population. Although a high degree of sexual mixing occurs between the ethnic groups, having sex with a partner of the same ethnic group was more common than would be expected if partner selection occurred regardless of ethnic group. Nevertheless, based on the MLST typing analysis there is no sound evidence for separate ethnic sexual transmission networks and differences in prevalence of chlamydia between ethnic groups could not be explained by ethnic sexual mixing patterns. Prevention activities must rather be targeted at the whole community at risk, with a focus on the younger age groups. Adequate testing facilities and subsequent treatment are needed to reduce the disease burden of chlamydia in Suriname.
